# Experimental Study on the Application of Cellulosic Biopolymer for Enhanced Oil Recovery in Carbonate Cores under Harsh Conditions

**DOI:** 10.3390/polym14214621

**Published:** 2022-10-31

**Authors:** Afeez Gbadamosi, Xianmin Zhou, Mobeen Murtaza, Muhammad Shahzad Kamal, Shirish Patil, Dhafer Al Shehri, Assad Barri

**Affiliations:** 1 Department of Petroleum Engineering, College of Petroleum and Geosciences, King Fahd University of Petroleum and Minerals, Dhahran 31261, Saudi Arabia; 2 Center for Integrative Petroleum Research, College of Petroleum and Geosciences, King Fahd University of Petroleum and Minerals, Dhahran 31261, Saudi Arabia

**Keywords:** enhanced oil recovery, biopolymer, viscosity, coreflooding, rheology, cellulose

## Abstract

Polymer flooding is used to improve the viscosity of an injectant, thereby decreasing the mobility ratio and improving oil displacement efficiency in the reservoir. Thanks to their environmentally benign nature, natural polymers are receiving prodigious attention for enhanced oil recovery. Herein, the rheology and oil displacement properties of okra mucilage were investigated for its enhanced oil recovery potential at a high temperature and high pressure (HTHP) in carbonate cores. The cellulosic polysaccharide used in the study is composed of okra mucilage extracted from okra (*Abelmoschus esculentus*) via a hot water extraction process. The morphological property of okra mucilage was characterized with Fourier transform infrared (FTIR), while the thermal stability was investigated using a thermogravimetric analyzer (TGA). The rheological property of the okra mucilage was investigated for seawater salinity and high-temperature conditions using a TA rheometer. Finally, an oil displacement experiment of the okra mucilage was conducted in a high-temperature, high-pressure core flooding equipment. The TGA analysis of the biopolymer reveals that the polymeric solution was stable over a wide range of temperatures. The FTIR results depict that the mucilage is composed of galactose and rhamnose constituents, which are essentially found in polysaccharides. The polymer exhibited pseudoplastic behavior at varying shear rates. The viscosity of okra mucilage was slightly reduced when aged in seawater salinity and at a high temperature. Nonetheless, the cellulosic polysaccharide exemplified sufficiently good viscosity under high-temperature and high-salinity (HTHS) conditions. Finally, the oil recovery results from the carbonate core plug reveal that the okra mucilage recorded a 12.7% incremental oil recovery over waterflooding. The mechanism of its better displacement efficiency is elucidated

## 1. Introduction

The current global energy crisis has shown that the significant contribution of oil and gas to the world energy supply is still irreplaceable. With growing calls to increase oil production, there is a need to develop novel techniques for recovering oil from depleted reservoirs [1]. Initial production from oil reservoirs is due to pressure depletion [2]. Thereafter, waterflooding is used to increase oil production [3]. Owing to immiscibility and density contrast, water tends to find the path of least resistance during flow in the reservoir, thereby causing the viscous fingering phenomenon [4]. Hence, a significant amount of oil is bypassed and left in the reservoir. To recover more oil from such reservoirs, numerous enhanced oil recovery (EOR) methods have been proposed. These can be broadly categorized into thermal and non-thermal EOR methods [5]. Thermal EOR methods require a huge amount of energy and water consumption, and cause large emissions into the environment [6]. Hence, non-thermal EOR methods are more commonly preferred [7].

Polymer flooding, a non-thermal EOR method, is adjudged to be highly effective owing to its efficiency in improving the macroscopic sweep efficiency of oil in reservoirs [8,9,10]. Polymeric solutions possess an inherent property of viscoelastic (viscous and elastic) effects [11]. Hence, they are used for EOR to recover bypassed oil thanks to their efficiency in increasing the viscosity of the injectant during flow in porous media [12]. Moreover, thanks to their viscoelasticity, polymers pull and strip oil in pore throats, thereby mobilizing oil trapped in pore scale ganglia towards the oil bank [13]. Besides, they cause disproportionate permeability reduction (DPR) by reducing the permeability of water [14]. Consequently, the mobility ratio of the displacing fluid to displaced fluid is decreased and a higher sweep efficiency is achieved.

The most widely used polymer for polymer flooding application is hydrolyzed polyacrylamide (HPAM) [9]. This is thanks to their good solubility and viscosifying property. Nevertheless, HPAM is less preferred in high salinity and high temperature conditions. At high salinity, the cation in the brine attacks the backbone of the polymer, which results in significant viscosity loss [15]. The viscosity loss is more significant in the presence of divalent ions. At a high concentration of divalent ions, precipitation of the polymer may occur [16]. Similarly, at high temperatures, hydrolysis of the polymeric backbone occurs and, consequently, loss of stability and viscosity of the polymeric solution is recorded [17]. Several attempts have been made to improve the viscosity of HPAM solution for harsh reservoir conditions. This includes the incorporation of salinity and temperature-resistant monomers and the application of nanotechnology. Nonetheless, the incorporation of the monomers increases the cost of the polymeric injectant while uncertainty persists in the toxicity nature of nano-additives [18]. Moreover, toxicity of synthetic polymers is a recent cause for concern.

Natural and biopolymers are gaining significant interest for their application in chemical EOR thanks to their eco-friendly nature [19,20,21]. Jang et al. [22] evaluated the efficiency of xanthan gum polymer for EOR under varying concentrations, shear rates, temperatures, and salinity. They observed that the biopolymer exhibited non-Newtonian behavior at high shear rates due to the alignment of the macromolecular chains along the line of flow. As compared with hydrolyzed polyacrylamide (HPAM), which witnessed significant viscosity reduction, xanthan polymer was found to withstand a high salinity concentration, while the viscosity decreased marginally under the influence of temperature. Oil displacement tests in a glass bead pack show that the application of xanthan gum for heavy oil recovery with 3 wt.% NaCl and 10 wt.% NaCl caused 28.4% and 30.1% additional oil recovery, respectively, over the waterflooding process. Similarly, Gao [23] noted that the field application of schizophyllan caused approximately 20% incremental oil recovery over the waterflooding process. Moreover, Xu et al. [24] observed that the application of welan gum demonstrated good rheological properties and caused a significant improvement in oil recovery compared with xanthan gum. Nonetheless, welan gum is unsuitable for high salinity and high temperature owing to anionic charges on the polymer backbone, while several complexities remain in understanding the rheological properties of schizophyllan [17,25].

**Figure 1:**
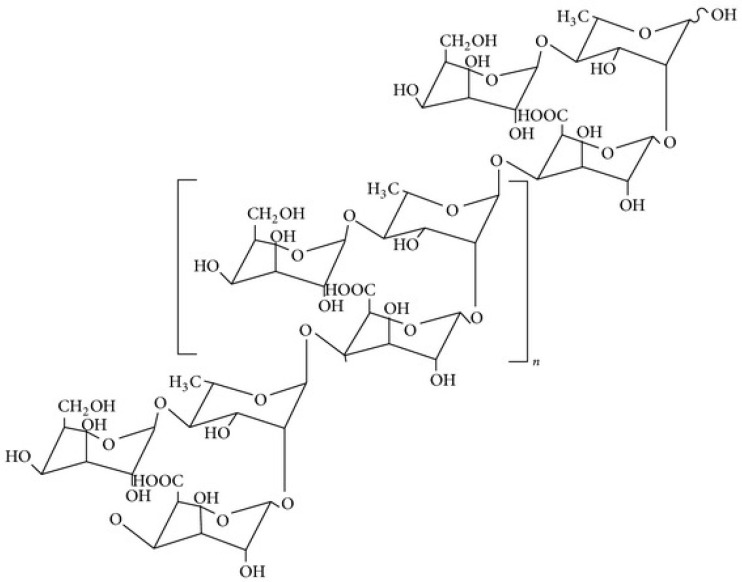
Structure of okra mucilage [29].

To overcome technical and toxicity concerns associated with synthetic polymers, okra mucilage (OM) was herein explored for its EOR potential. Okra mucilage is a green extract from okra fruits [26,27]. Okra belongs to the family of cellulosic polysaccharides (Figure 1). The mucilage demonstrates viscoelastic properties, which are desirable for use as a polymer injectant [28]. Several methods are used for the extraction of mucilage from okra. This includes but is not limited to microwave-assisted extraction, hot water extraction, ultrasonic extraction, and the pressurized water extraction process [26]. Herein, mucilage was extracted from okra via a hot water extraction process because it is a non-destructive technique. The extracted mucilage was characterized using Fourier infrared transform (FTIR) and a thermogravimetric analyzer (TGA). The rheological properties of the extracted okra mucilage (OM) were measured at varying salinities and temperatures. Finally, the application of OM as a polymeric solution for the tertiary recovery of oil from carbonate cores was studied at high temperatures and high pressure.

## 2. Materials and Methods

### 2.1. Materials

**Figure 2:**
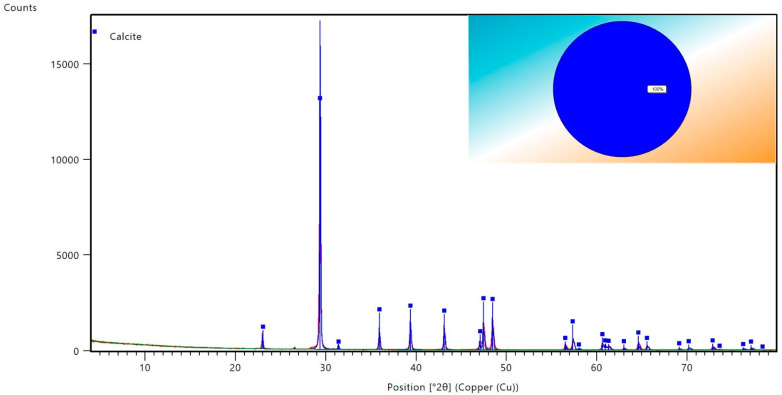
X-ray diffraction analysis of the carbonate core sample.

Okra pods used for the experiment were bought from the local market. Sodium chloride (NaCl), calcium chloride (CaCl_2_), magnesium chloride (MgCl_2_), sodium sulfate (Na_2_SO_4_), and sodium hydrogen carbonate (NaHCO_3_) were purchased from Sigma Aldrich. The salts were used for preparing seawater and formation water with the composition depicted in Table 1. Deionized (DI) water obtained from Milli-Q was used for preparing the solution throughout the experiment. Indiana limestone carbonate cores were used with the properties presented in Table 2. X-ray diffraction analysis of the core is depicted in Figure 2. The SARA components and other properties of the crude oil are also presented in Table 3.

### 2.2. Preparation of Okra Polysaccharide

Prior to use, the okra was thoroughly washed with DI water to remove impurities and sliced. Thereafter, the hot water extraction process was used to obtain mucilage from the sliced okra. The sliced okra was heated with seawater at 40 °C for 30 min. All experiments were conducted using seawater because the experiment is interested in the performance of OM polymer under high salinity conditions. The mucilage was removed from the okra pod by decantation and subsequently filtered using a muslin cloth. Thereafter, the obtained mucilage was centrifuged and the supernatant collected was cooled under ambient conditions and stored in a glass bottle under an inert atmosphere. The yield was estimated using Equation (1).

(1) $$Yield\;\left(\%\right)=\frac{W_{ce}}{W_{t}}\times 100\%$$

where $W_{ce}$ is the weight of the cellulosic extract and $W_{t}$ is the total weight of the dissolved okra.

The solubility of OM was determined via centrifugation of a known concentration of OM at 3000 rpm for 20 min. The supernatant was removed and the settled portion was dried on a petri dish in an oven at 100 °C. The solubility was determined using Equation (2).

(2) $$Solubility\;\left(\%\right)=\frac{C_{i}-C_{f}}{C_{i}}\times 100\%$$

where *C_i_* is the initial concentration of the solution (mg) and *C_f_* is the fraction segmented (mg).

### 2.3. Morphological Characterization

Fourier transform infrared (FTIR) was conducted to determine the morphological properties of OM. The structural features were measured using the Bruker INVENIO FTIR spectrophotometer. To determine the thermal stability, the thermogravimetric analyzer (TGA) of the sample was measured over a wide range of temperatures from 25 to 700 °C using PerkinElmer TL 8000.

### 2.4. Rheological Test

The rheological property of the polymers in seawater salinity at varying temperatures was measured using a TA rheometer. The rheometer is equipped with a water bath for adjusting the temperature of the equipment to the desired range. The steady shear of OM was measured over the range of 0.1–1000 $s^{-1}$.

### 2.5. Oil Displacement Test

**Figure 3:**
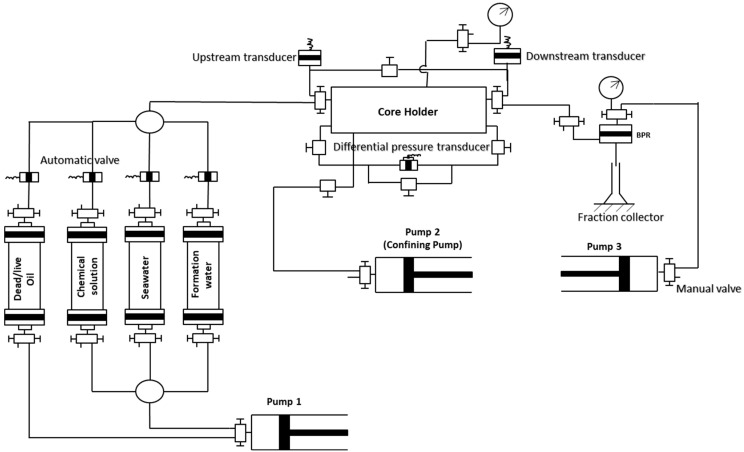
Schematic of the core flooding setup.

To evaluate oil recovery after injecting polymer, an oil displacement experiment was carried out using high-pressure, high-temperature (HPHT) core flooding equipment. Firstly, the cores were polished and dried. Afterward, the porosity was measured using a Helium porosimeter manufactured by Vinci technologies. Prior to loading the carbonate core samples in the equipment, the cores were saturated. Subsequently, the permeability of the cores was determined using a liquid permeameter manufactured by Vinci technologies. The core flooding equipment consists of an oven for adjusting the temperature to the desired range and four accumulators for placing the oil, seawater, formation water, and OM polymer. The core was loaded into the core holder and the confining pressure of 2500 psi was applied. Afterward, a back pressure of 1500 psi was applied using the backpressure regulator (BPR) to maintain the overburden pressure during core flooding. The system was heated to 80 °C. Formation water was injected to saturate the core. Subsequently, oil was injected to establish the initial water saturation ($S_{wi}$). The system was left for 48 h to achieve equilibrium. Thereafter, seawater was injected to mimic the water flooding phenomenon. The seawater was injected at a flow rate of 0.5 cc/min and the oil recovered was recorded. After water flooding, OM was injected to estimate oil recovery. The schematic of the oil displacement process is shown in Figure 3.

## 3. Results and Discussion

### 3.1. Extraction and Characterization of OM

**Figure 4:**
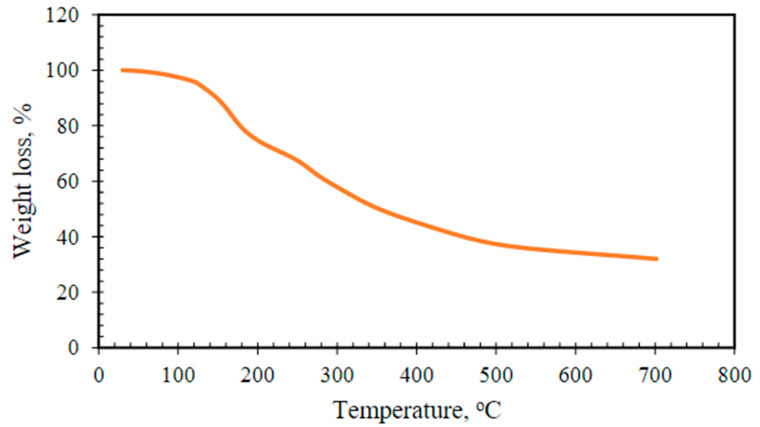
Thermal stability analysis of OM polymer via TGA [30].

**Figure 5:**
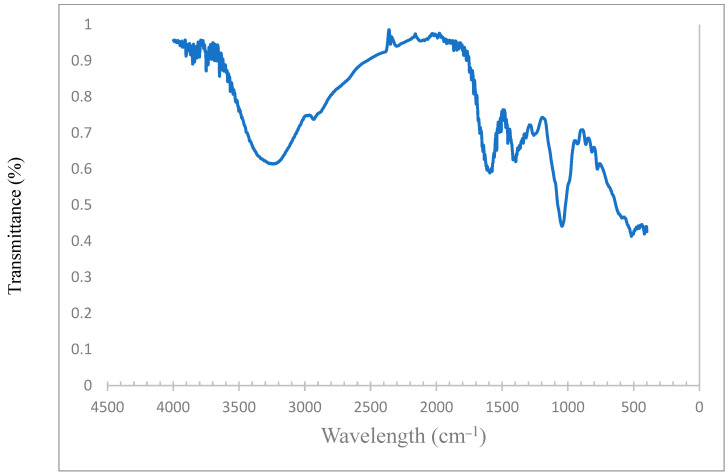
FTIR spectra of okra mucilage.

The yield of the extracted cellulosic OM polysaccharide is estimated as 9.4 *w/w*%. This agrees with previous report, which noted that the OM yield for the hot water extraction process of okra varies from 0.5 to 15% *w/w* [26,27]. The solubility of OM was determined to be approximately 90% at a pH of 6.2. Figure 4 illustrates the thermal stability analysis of the OM polymer from 25 to 700 °C. Three stages of weight loss were recorded over the entire temperature range measured. Firstly, weight loss was encountered at around 100 °C, which may be attributed to the loss of water (H_2_O) content of the polymeric sample. Afterwards, weight loss was recorded in the temperature range of 170–250 °C, which may be ascribed to the decomposition of the polysaccharide in the OM polymer. Finally, loss of carbon residue caused weight loss in the temperature range of 250–450 °C. Furthermore, the morphological property of OM polymer was examined using an FTIR spectrophotometer, and the transmittance range is shown in Figure 5. The OM consists of galactose, rhamnose, and galacturonic acid constituents. A peak was observed at 3335 cm^−1^, which may be attributed to the hydroxyl (–OH) functional group. Moreover, a peak was captured at approximately 2900 cm^−1^, which represents the stretching vibration of the –C–H bond constituent of the galactose and rhamnose. Another peak was recorded at 1720 cm^−1^, which may be ascribed to the –C=O bond of the galacturonic acid.

### 3.2. Rheological Behavior of OM Polymer

**Figure 6:**
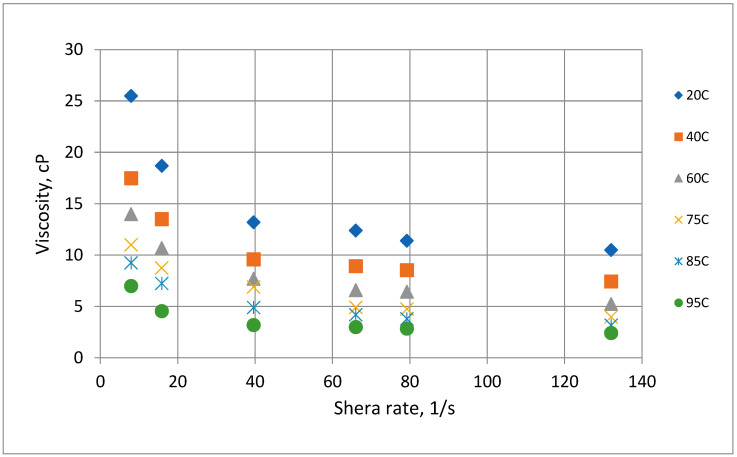
Viscosity of OM polymer versus shear rate with different testing temperatures in seawater.

**Figure 7:**
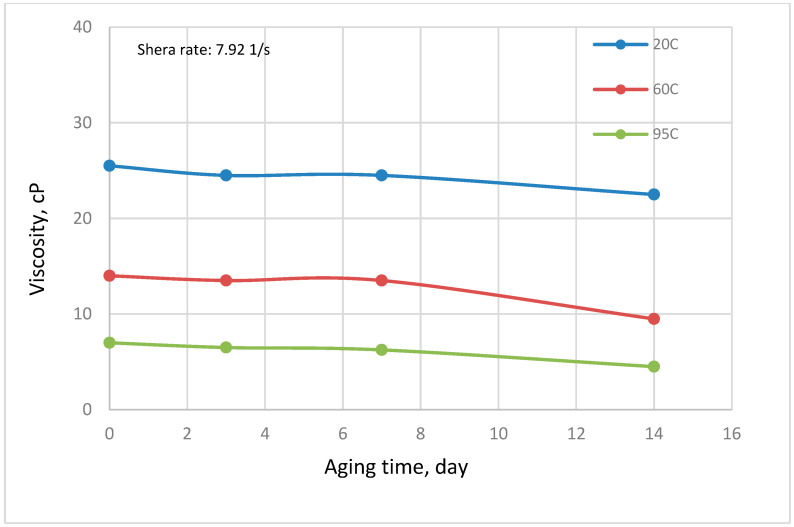
Effect of aging time on the rheological behavior of OM in seawater salinity (temperature = 20, 60, and 95 °C; shear rate = 7.92 s^−1^).

**Figure 8:**
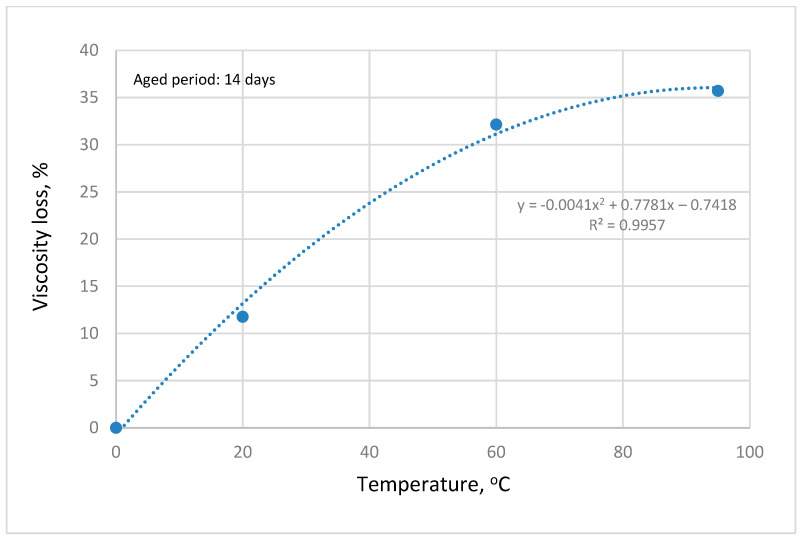
Viscosity loss of OM in seawater during the aging period of 14 days with different temperatures.

The rheological behavior of OM in seawater salinity as a function of temperature and shear rate is depicted in Figure 6. OM polymer is composed of an array of cooperative non-covalent bonds in its macromolecular structure. The polymer demonstrated pseudoplastic behavior whereby the viscosity of the OM polymer decreases with an increase in shear rate. At low shear rates, the viscosity of OM polymer is sufficiently high because of the ordered structural configuration of the polysaccharide chain entanglements. On the other hand, the polymer recorded a low viscosity at high shear rates owing to the stretching of the OM and disruption of the polymeric chain network by the large shear forces [28]. Similarly, an increase in the temperature of the solution reduced the viscosity of the polymer molecules. This can be attributed to the thermal motion, which resulted in the disentanglement of the polymeric chain [31]. To study the stability of OM polymer, the polymeric solution was aged at different temperatures for several days and the viscosity was constantly measured. As illustrated in Figure 7, the thermal aging of the polymer causes a reduction in viscosity, though quite infinitesimal. The percentage viscosity reduction for a 14-day period is presented in Figure 8. Aging at 95 °C caused the most significant viscosity loss of 35% compared with aging at an ambient temperature, which resulted in a paltry viscosity loss of around 12%. The viscosity loss under ambient conditions and a high temperature over the entire time can be ascribed to biodegradation of the polymeric molecules. Nonetheless, despite the viscosity loss, OM polymeric molecules showed no sign of precipitation, which may hinder their transport in porous media. Moreover, the viscosity recorded is sufficient to reduce the mobility of injected waterflood and enhance the macroscopic sweep efficiency. 

### 3.3. Oil Displacement Test

**Figure 9:**
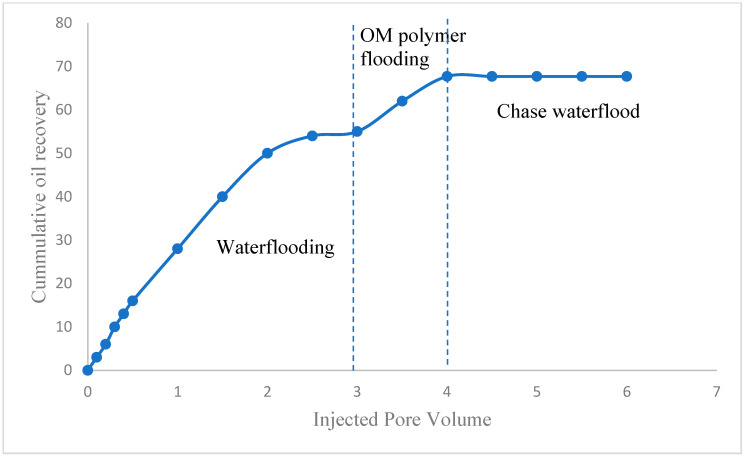
Cumulative oil recovery versus injected pore volume.

The performance evaluation of OM as an EOR agent was conducted under HPHT conditions of 2500 psi and 80 °C. Figure 9 depicts the oil recovery as a function of the pore volume of fluids injected. The injection of waterflood recovered 55% of the original-oil-in-place (OOIP). A significant proportion of oil is bypassed and left behind in the carbonate cores. This can be attributed to the viscous fingering phenomenon, which causes the injected water flood to finger through the path of least resistance of high permeability channels, thereby leaving behind a significant proportion of the oil. Subsequently, polymer flooding was commenced by injection of OM polymer as an EOR injectant. OM polymer caused an incremental oil recovery of approximately 12.7% OOIP. The injection of OM polymer causes an increase in the viscosity of the injectant, which consequently decreases the mobility of the injectant. Decreased mobility of the injectant enables it to push bypassed oil because of its viscoelastic property. Moreover, the OM polymer may cause a disproportionate permeability reduction by plugging high permeability channels and diverting subsequently injected waterflood to low permeability channels to recover more oil from the reservoir.

## 4. Conclusions

This study evaluates the oil displacement behavior of OM polymer for EOR after the conventional secondary recovery process. The FTIR result shows that OM polymer possesses galactose, rhamnose, and galacturonic acid constituents, while the TGA result confirms the stability of the polymer over a wide range of temperatures. OM polymer exhibited shear thinning behavior when subjected to shear forces. The polymer demonstrates relatively good stability when aged at a high temperature for 2 weeks. The waterflooding process caused the recovery of 55% OOIP. On the other hand, the injection of OM polymer in seawater salinity and a high temperature (80 °C) resulted in the recovery of an additional 12.7% OOIP. Thanks to its efficiency under high salinity and high temperature conditions, OM polymer is proffered for use as an EOR agent in carbonate reservoirs.

## Figures and Tables

**Table 1:** Composition of seawater and formation water.

Salt	Seawater (g/L)	Formation Water (g/L)
NaCl	41.172	150.446
CaCl_2_·2H_2_O	2.387	69.841
MgCl_2_·6H_2_O	17.644	20.396
Na_2_SO_4_	6.339	0.518
NaHCO_3_	0.165	0.487
TDS	67.71 g/L	241.688 g/L

**Table 2:** Properties of carbonate core samples.

Properties	Unit
Diameter	1.5″
Length	6.03″
Porosity	18.47
Permeability	25.5 mD

**Table 3:** Properties of crude oil.

Properties	Values
Saturates	36.2
Aromatics	50.0
Resins	11.0
Asphaltene	2.8
Density	0.862 g/cm^3^
Viscosity	10.9 cP

## Data Availability

Data available based on request.
